# A Novel Method for Estimating and Balancing the Second Harmonic Error of Cylindrical Fused Silica Resonators

**DOI:** 10.3390/mi12040380

**Published:** 2021-04-01

**Authors:** Yunfeng Tao, Yao Pan, Jianping Liu, Yonglei Jia, Kaiyong Yang, Hui Luo

**Affiliations:** College of Advanced Interdisciplinary Studies, National University of Defense Technology, Changsha 410073, China; taoyunfeng13@nudt.edu.cn (Y.T.); l_jianp@sina.com (J.L.); jiayonglei17@nudt.edu.cn (Y.J.); yky208@nudt.edu.cn (K.Y.); luohui.luo@163.com (H.L.)

**Keywords:** cylindrical resonator, chemical balancing, frequency split, second harmonic error

## Abstract

The cylindrical resonator gyroscope (CRG) is a type of Coriolis vibratory gyroscope which measures the angular velocity or angle through the precession of the elastic wave of the cylindrical resonator. The cylindrical fused silica resonator is an essential component of the CRG, the symmetry of which determines the bias drift and vibration stability of the gyroscope. The manufacturing errors breaking the symmetry of the resonator are usually described by Fourier series, and most studies are only focusing on analyzing and reducing the fourth harmonic error, the main error source of bias drift. The second harmonic error also is one of the obstacles for CRG towards high precision. Therefore, this paper provides a chemical method to evaluate and balance the second harmonic error of cylindrical fused silica resonators. The relation between the frequency split of the *n* = 1 mode and the second harmonic error of the resonator is obtained. Simulations are performed to analyze the effects of the first three harmonic errors on the frequency splits. The relation between the location of the low-frequency axis of *n* = 1 mode and the heavy axis of the second harmonic error is also analyzed by simulation. Chemical balancing experiments on two fused silica resonators demonstrate the feasibility of this balancing procedure, and show good consistency with theoretical and simulation analysis. The second harmonic error of the two resonators is reduced by 86.6% and 79.8%, respectively.

## 1. Introduction

The Coriolis vibratory gyroscope measures angular velocities or angles by the precession of the standing wave [1]. The cylindrical resonator gyroscope (CRG) and the hemispherical resonator gyroscope (HRG) are two types of Coriolis gyroscopes, and they are widely used in inertial navigation systems. HRGs have unparalleled precision guaranteed by a highly symmetrical resonator with extremely small dissipation. The HRG Crystal^TM^ DUAL CORE made by Safran Electronics & Defense (Massy, France) has achieved a bias stability of 0.0001°/h over 100 h [2]. Inspired by these breakthroughs, HRGs have become a topic of interest within the inertial community, with devoted researchers investigating traditional size HRGs [3,4,5,6] and micro-version HRGs [7,8,9]. However, the complexity of the manufacturing of HRGs has limited the mastering of this technology to a small number of manufacturers.

Compared to HRGs, the CRGs are simpler to manufacture. Similar to HRGs, the key component of a CRG is the cylindrical resonator, the quality factor and the residual imperfections of which generally determine the performance of the CRG. Many researchers have been devoted to improving the quality factor of cylindrical resonators through the selection of resonator material, optimizing the structure, improving the processing technology and applying special post-processing treatments [10,11,12,13]. Based on these improvements, the quality factor of cylindrical fused silica resonator has achieved 2.89 million and still improving [14], which is sufficient for medium precision applications and makes CRGs a promising candidate for tactical platforms and civil applications. 

Although the cylindrical resonator is easier to manufacture compared with the hemispherical resonator, the machining defects are always inevitable. These defects, especially the asymmetrical distribution of mass and density, will cause the damping to differ from location to location on resonator and the ideal single natural frequency to split [15]. The circumferentially distributed imperfections originated from machining can be described with Fourier series [16]. For the *n* = 2 mode, the first four harmonic errors of the Fourier series will severely destroy the standing wave on the resonator. The fourth harmonic error mainly leads to the frequency split of the *n* = 2 mode and the preferred angular location of the standing wave, which results in the orthogonal error and bias drift of the resonator gyroscope [17]. The first three harmonic errors lead to the undesirable oscillations, which distort the detection signal. The undesirable oscillations are equivalent to the addition of central angle-dependent damping to the vibration system. Consequently, the quality factor varies with different central angles [18]. Therefore, the first four harmonic errors must be decreased as much as possible within the limits of balancing methods to achieve higher performance of CRGs. 

Among the first four harmonic errors, the fourth harmonic error is extensively studied. The fourth harmonic error mainly causes the frequency split of the *n* = 2 mode and is a primary error source of the gyroscope. There are several ways to reduce the fourth harmonic error, such as electrostatic trimming [19,20,21], mechanical trimming [22,23,24], femtosecond laser trimming [25], chemical trimming [26,27], and ion beam trimming [28,29]. The electrostatic trimming requires extra voltages which makes the circuit more complicated and may introduce new noise and errors. The mechanical trimming may cause new defect during the mechanical drilling and grinding. The laser trimming produces thermal stress during the high-energy laser interacting with the resonator. The thermal stress leads to the decreasing of the quality factor. Ion beam trimming can remove materials from the resonator precisely. However, it is not suitable for the trimming of large frequency split. The frequency split of the *n* = 2 mode can be reduced to mHz level using the appropriate trimming technique.

The first three harmonic errors are a significant factor causing the deterioration of the gyroscope performance. However, significantly little research has focused on the theoretical aspects, identification methods and balancing techniques of the first three harmonic errors. The precise determination of the first three harmonics requires complicated measuring systems, and it is rather difficult to measure the value and direction of the first three harmonics precisely. Therefore, attempts to explore the measuring and balancing methods for the first three harmonic errors are rather rare. This study intends to focus on theoretical aspects, measurement methods, and balancing methods of the second harmonic error. By understanding the relationship between the first three harmonic errors with the symmetric flexural modes of the resonator, we proposed a novel identification method for the second harmonic error. Based on the balancing of the fourth harmonic error by chemical etching in our previous work [30], we made a further modification to the chemical trimming theory. The modified chemical trimming theory for the balancing of the second harmonic error was experimentally verified. The performance of the resonator is further improved after the chemical balancing of the second harmonic error.

This paper is organized as follows: Section 2 analyzes the influence of the unbalanced mass on the quality factor. Section 3 deduces the relation between the frequency split of *n* = 1 mode and the second harmonic error of the imperfect density. The impacts of the first three harmonic errors on the frequency split of the *n* = 1, 2, and 3 modes is evaluated by FEM analysis. Section 4 extends the chemical trimming method we applied for the trimming of the *n* = 2 mode to the balancing of the second harmonic error and investigates the dependence of the low-frequency axis of *n* = 1 mode on the heavy axis. Chemical balancing experiments on two cylindrical fused silica resonators are demonstrated in Section 5, followed by remarks and conclusion in Section 6. 

## 2. The Influence of the Unbalanced Mass on Quality Factor

A cylindrical resonator with a quality factor around 5 million is adequate for the performance up to 0.01°/h. The real obstacle in the way of inertial grade cylindrical resonator gyroscopes comes from the unbalanced mass. As the first three harmonics of the unbalanced mass lead to motion of the center of mass, the real quality factor depends on the properties of the connection between the resonator and the base [31]. The quality factor *Q_u_* induced by the unbalanced mass is given by:

(1) $$\frac{1}{Q_{u}}=\frac{1}{2\pi }\frac{m_{u}}{M_{d}}\arctan\left(\frac{\omega_{h}\times \omega_{2}}{Q_{h}\left(\omega_{h}^{2}-\omega_{2}^{2}\right)}\right),$$

where *m_u_* is the unbalanced mass of the resonator, *M_d_* is the modal mass of the resonator, *ω_h_* and *Q_h_* is the frequency and the quality factor of the harmonic mode respectively, *ω*_2_ is the natural frequency of the *n* = 2 mode.

According to Equation (1), the unbalanced mass and the characteristics of fixation between resonator and base both affect the quality factor *Q_u_*.

The real quality factor *Q_r_* for a assembled resonator is then:

(2) $$\frac{1}{Q_{r}}=\frac{1}{Q_{n}}+\frac{1}{Q_{u}},$$

where *Q_n_* is the nominal quality factor of the *n* = 2 mode. 

For the resonator with nominal quality factor *Q_n_* over one million, the real quality factor *Q_n_* approximately equal to unbalanced quality factor *Q_u_*. The position of the standing wave hinges on the central angle of the second harmonic error under external vibrations, and the increasing rate of the vibration amplitude of the standing wave is proportional to the value of the second harmonic error. The value and direction of the first and the third harmonics can be identified by measuring the amplitude and the central angle of the standing wave under transverse vibration of the base [32]. Due to the presence of the first three harmonic errors, the resonator gyroscope is sensitive to external vibrations causing the deterioration in the bias drift and random walk.

## 3. Theory and Simulation Aspects of the Second Harmonic Error on Frequency Split

### 3.1. Relationship between the Second Harmonic Error and the Frequency Spilt of the n = 1 Mode

The effective sensing element of a cylindrical resonator is the resonator shell. Therefore, the ring model is applied to describe the characteristics of a cylindrical resonator. 

**Figure 1:**
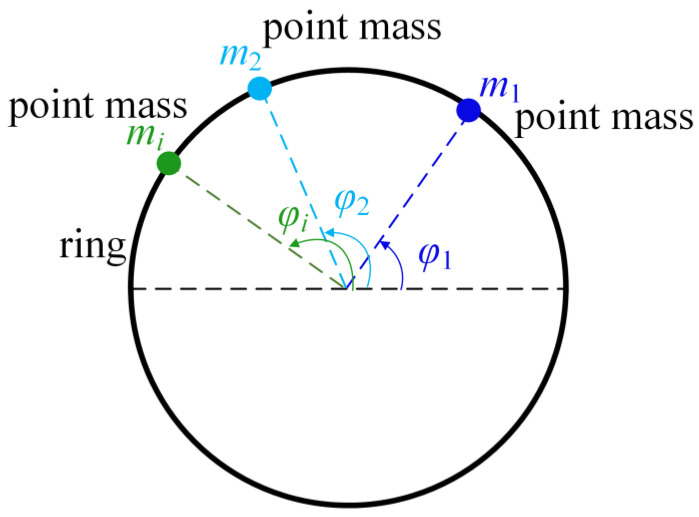
The ring with attached mass points.

The cross-section of a thin circular ring with axial length *L* is shown in Figure 1. The mass point *m_i_* is attached at the central angle *φ_i_*. The imperfections of the resonator are modeled by the circumferentially varied density. The density of a resonator with imperfections can be expressed by Fourier series and only take the first three harmonic errors in the case:

(3) $$\rho \left(\varphi \right)=\rho_{0}+\rho_{1}\cos\left(\varphi \right)+\rho_{2}\cos\left(2\varphi \right)+\rho_{3}\cos\left(3\varphi \right)+\cdots ,$$

where *ρ* is the equivalent density of the imperfect resonator, *ρ*_0_ is the density of the perfect resonator, *ρ_i_* is the *i*-th harmonic error of the imperfections, and *φ* is the central angle.

The natural frequency of the cylindrical resonator in the *n*-th mode is given by [33]:

(4) $$\omega_{n1,n2}^{2}=\omega_{n0}^{2}/{\left[1+\sum_{i}m_{i}/M_{0}\pm \frac{\alpha_{n}^{2}-1}{M_{0}\left(\alpha_{n}^{2}+1\right)}\left({\left(\sum_{i}m_{i}\cos2n\varphi_{i}\right)}^{2}+{\left(\sum_{i}m_{i}\sin2n\varphi_{i}\right)}^{2}\right)\right]}^{1/2},$$

where *ω_n_*_1_, *ω_n_*_2_ is the natural frequency of the *n*-th mode for an imperfect resonator, *ω_n0_* is the natural frequency of a perfect resonator, *m_i_* is the attached point mass, *φ_i_* is the central angle of point mass *m_i_*, *M*_0_ is the mass of the perfect resonator, and *α_n_* is the amplitude ratio of the radial and the tangential vibration. 

The frequency split is defined as the frequency difference between the natural frequencies of the *n*-th mode. Therefore, the frequency split can be calculated from Equation (4):

(5) $$\begin{array}{l} \Delta \omega_{n}=\omega_{n1}-\omega_{n2}={\left[{\left(\sum_{i}m_{i}\cos2n\varphi_{i}\right)}^{2}+{\left(\sum_{i}m_{i}\sin2n\varphi_{i}\right)}^{2}\right]}^{1/2}\omega_{n0}\left(\alpha_{n}^{2}-1\right)/\left[M_{0}\left(\alpha_{n}^{2}+1\right)\right], \end{array}$$

where Δ*ω_n_* is the frequency split of the *n*-th mode in rad/s.

As the number of discrete mass points increases, the mass defect can be expressed as an integration over the central angle *φ*:

(6) $$\Delta \omega_{n}={\left[{\left(\int_{0}^{2\pi }\rho \left(\varphi \right)\cos\left(2n\varphi \right)d\varphi \right)}^{2}+{\left(\int_{0}^{2\pi }\rho \left(\varphi \right)\sin\left(2n\varphi \right)d\varphi \right)}^{2}\right]}^{1/2}\omega_{n0}\left(\alpha_{n}^{2}-1\right)/\left[M_{0}\left(\alpha_{n}^{2}+1\right)\right].$$

Substituting Equation (3) into Equation (6), we can obtain the relationship between *ρ*_2_ and the frequency split of the *n* = 1 mode Δ*ω*_1_:

(7) $$\Delta \omega_{1}=\omega_{10}\left(\alpha_{1}^{2}-1\right)\left|\rho_{2}\right|\pi /\left[M_{0}\left(\alpha_{1}^{2}+1\right)\right].$$

Note that the frequency split of the *n* = 1 mode Δ*ω*_1_ is proportional to the second harmonic error *ρ*_2_. In other words, we can reduce the second harmonic error by frequency tuning of the *n* = 1 mode. Once the frequency split of the *n* = 1 mode decreases, the second harmonic error decreases accordingly. The frequency split of the *n* = 1 mode is readily measurable by the system developed for the measurement of the *n* = 2 mode, therefore, this new method avoids developing a special measurement system for the determination of the second harmonic error by vibration coupling. This novel method provides an effective and practical approach to identify the second harmonic error of the resonator which makes the balancing possible. The frequencies of the resonator in the FEM analysis and our experiments were given in Hz, therefore, we calculate the frequency split of the *n*-th mode Δ*f_n_* in Hz, and Δ*f_n_* = Δ*ω_n_*/2π.

### 3.2. FEM Analysis of the First Three Harmonic Errors

**Figure 2:**
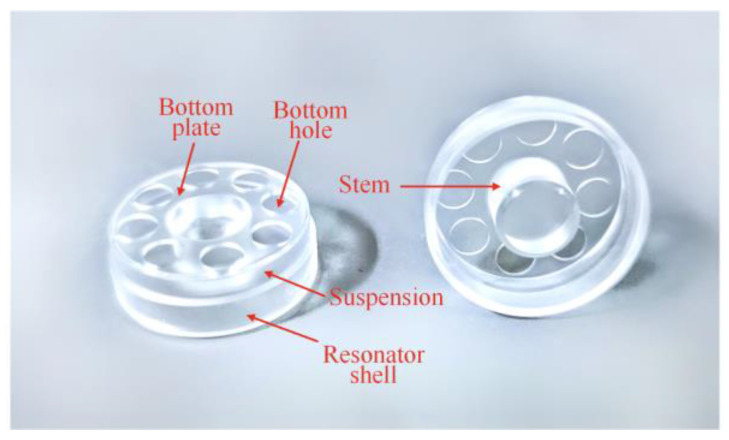
The description of the cylindrical fused silica resonator.

It is widely recognized that the 2*k*-th harmonic error mainly contributes to the frequency split of the *n* = *k* mode. FEM analysis on our structured resonators is employed to investigate the quantitative relationship between the first three harmonic errors and the *n*-th (*n* = 1, 2, 3) mode. The half-section view of the cylindrical fused silica resonator is shown in Figure 2. The resonator shell is the main sensing element whose imperfections have a significant impact on the gyroscope performance. The suspension and the bottom plate is the elastic suspension, the stiffness of which can be adjusted by changing the thickness or the size of the eight holes on the bottom plate for different designs. The inner stem is used to mount the resonator on a base [12].

The parameters of the material and the structure of the resonator are listed in Table 1.

**Figure 3:**
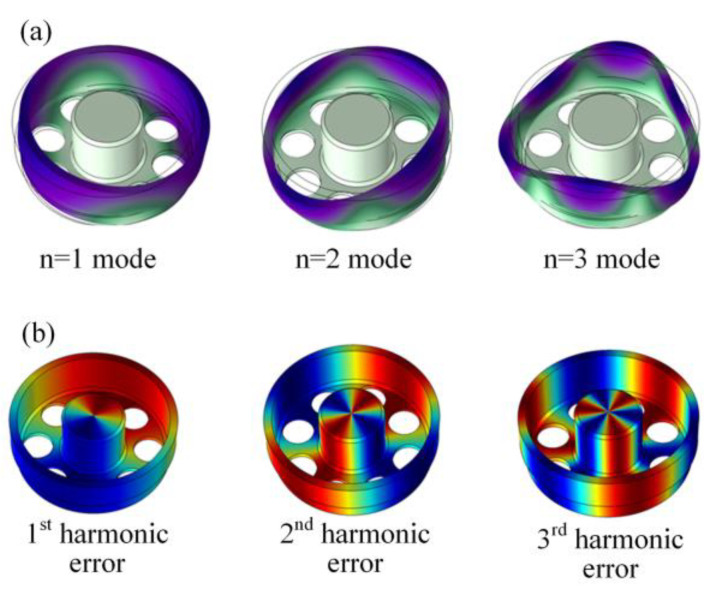
(**a**) The first three vibration modes of a cylindrical resonator. (**b**) The distribution of the first three nonuniform density when Δ*ρ_i_* = 0.05%.

The modes of vibration of a resonator without density errors is simulated, and the *n* = 1, 2 and 3 modes are shown in Figure 3a. Note that even the resonator without density errors has a frequency split around 0.1 Hz due to the mesh error of an irregular bottom plate. This frequency split is caused by the quality of the grids in the FEM model. Therefore, the zero-closed frequency splits which smaller than 0.1 Hz stems from the grids instead of the harmonic errors.

According to Equation (3), the density of the resonator is set as a continuous function of central angle *φ* in the FEM model. To investigate the impact of the first three harmonic errors on frequency splits separately, three functions of nonuniform density are employed as follows:

(8) $$\rho \left(\varphi \right)=\rho_{0}+\rho_{i}\cos\left(i\varphi \right)\;\;\;i=1,2,3.$$

The degree of imperfections Δ*ρ_i_* is *ρ_i_*/*ρ*_0_. The circumferential distribution of imperfect density on resonator when Δ*ρ_i_* = 0.05% is given in Figure 2. The distribution cycle of density with the first three harmonic errors is 2π, π, and 2π/3, respectively.

**Figure 4:**
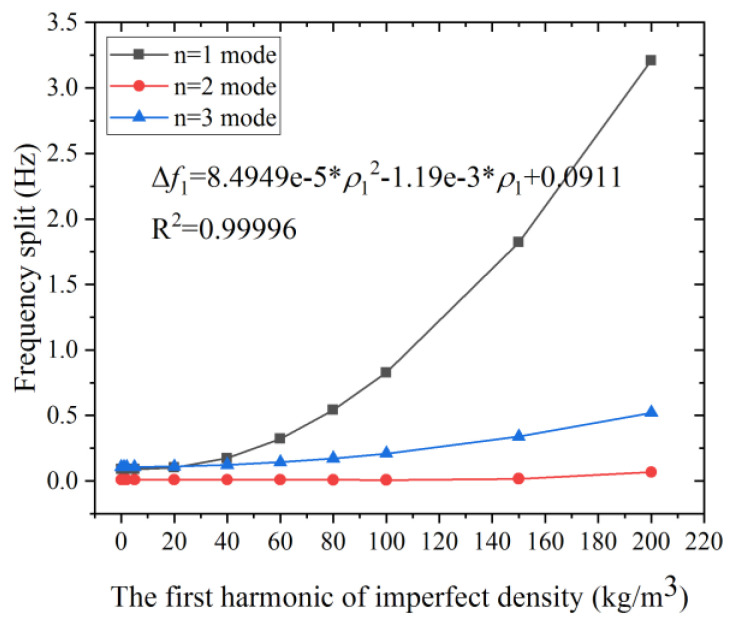
The effect of *ρ*_1_ on the frequency split of the *n* = 1, 2, and 3 modes.

The effects of the first three harmonics of density on the frequency split of the *n* = 1, 2, and 3 modes are investigated. As shown in Figure 4, the frequency split of the *n* = 1 and the *n* = 3 mode increases with the increasing first harmonic error, while the frequency split of the *n* = 2 mode remains small (around 0.0095 Hz) when the value of imperfect density is lower than 100 kg/m^3^ and Δ*ρ_i_* = 4.5%. Moreover, the growth rate of the frequency split of the *n* = 1 mode (shown in grey line) is much faster than that of the *n* = 3 mode (shown in blue line). The first harmonic error has a larger impact on *n* = 1 mode than the other two, and the frequency split of *n* = 1 mode is approximately proportional to the square of the first harmonic error when the second harmonic error is more than 15 kg/m^3^, as shown in the curve fit of Δ*f*_1_. 

**Figure 5:**
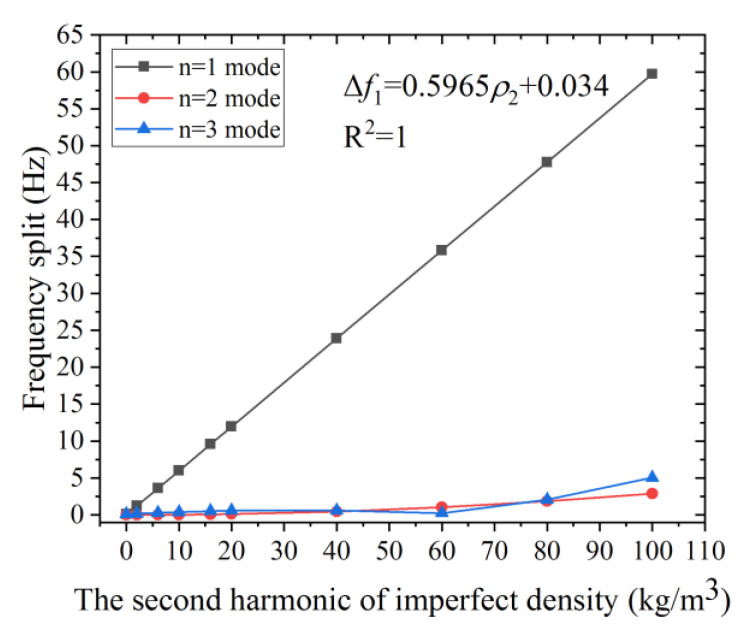
The effect of *ρ*_2_ on the frequency split of the *n* = 1, 2, and 3 modes.

Figure 5 shows that the frequency split of the *n* = 1 mode increases rapidly with the growth of the second harmonic error, while that of the *n* = 2 and the *n* = 3 mode increase only mildly. For example, the frequency split of *n* = 1 mode reaches 38.67 Hz when *ρ*_2_ is 65 kg/m^3^, while the frequency split of the *n* = 2, 3 mode is only 1.19 Hz and 0.99 Hz, respectively. The frequency split of *n* = 1 mode is severely affected by *ρ*_2_ compared with the other two modes, even when the defect is small. Moreover, Δ*f*_1_ grows linearly with the increase of *ρ*_2_, as illustrated by the grey line and the least-square fit. Therefore, this simulation result is consistent with (5). Nearly 95% of the frequency split Δ*f*_1_ comes from the second harmonic error when Δ*ρ_2_* is 2.95%. The frequency split Δ*f*_1_ can be used for the evaluation of the second harmonic error. As we obtain the frequency split Δ*f*_1_, the value of the *ρ_2_* can be calculated from the fitted formula. 

**Figure 6:**
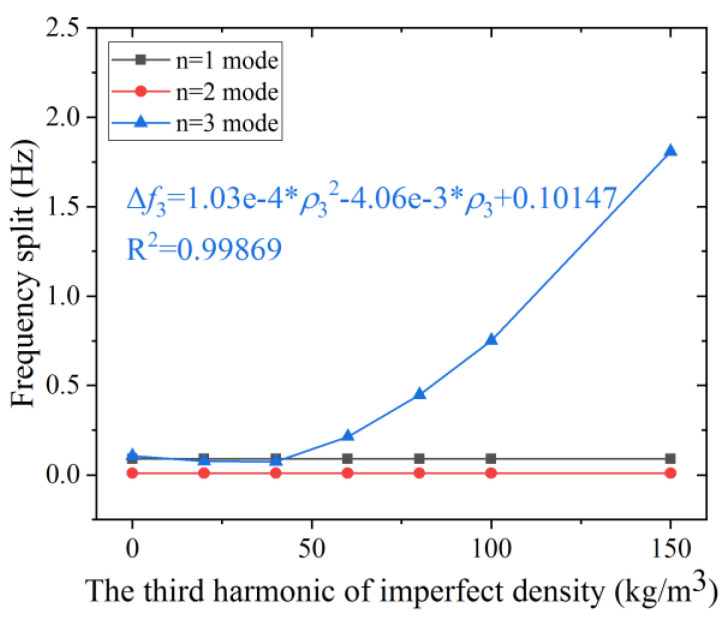
The effect of *ρ*_3_ on the frequency split of the *n* = 1, 2, and 3 modes.

The simulation result shown in Figure 6 indicates that the frequency split of the *n* = 3 mode is proportional to the square of the third harmonic error. The influence of *ρ*_3_ on the *n* = 1, 2 mode is rather small compared that on the *n* = 3 mode. The frequency split of the *n* = 1 mode keeps almost unchanged at 0.089 Hz. And the frequency split of *n* = 2 mode stay constant at 0.0096 Hz. Therefore, it is reasonable to consider that the third harmonic error has no impact on the frequency split of the *n* = 2, 3 mode. The frequency split of *n* = 3 mode is approximately proportional to the square of the third harmonic error when the second harmonic error is more than 40 kg/m^3^, as shown in the curve fit of Δ*f*_3_. 

**Figure 7:**
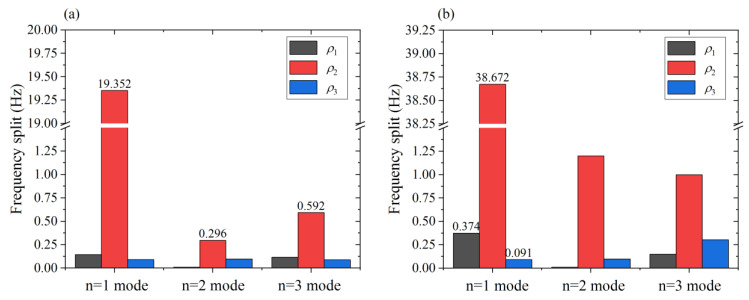
Frequency split of the *n* = 1, 2, and 3 modes: (**a**) When the harmonic error *ρ_i_* = 32 kg/m^3^; (**b**) When the harmonic error *ρ_i_* = 65 kg/m^3^.

Figure 7 depicts the impact of *ρ_i_* on the frequency split Δ*f_n_* with the same magnitude of the harmonic error. It can be seen in Figure 6 that the second harmonic error has the most significant impact on the frequency split of all three modes, especially the *n* = 1 mode. In fact, the second harmonic error accounts for the largest proportion of Δ*f*_1_. The Δ*ρ_i_* is usually small, no larger than 5%. As shown in Figure 6, the frequency split of the three modes is 19.352 Hz, 0.296 Hz and 0.592 Hz, respectively, when the second harmonic error is 32 kg/m^3^ (where Δ*ρ*_2_ is 1.45%). In contrast, the first and the third harmonic error have little influence on Δ*f*_1_. The frequency split Δ*f*_1_ caused by the second harmonic error is 133 times and 215 times that of the first and third harmonic error when Δ*ρ_i_* is 1.45%. As the harmonic error reaches 65 kg/m^3^ (where Δ*ρ_i_* is 2.95%), the frequency split Δ*f*_1_ under harmonic errors *ρ*_1_, *ρ*_2_, and *ρ*_2_ is 0.374 Hz, 38.672 Hz and 0.091 Hz, respectively. Hence, *ρ*_1_ and *ρ*_3_ make far less contribution to Δ*f*_1_ compared with *ρ*_2_. Therefore, it is convenient to determine the value of the second harmonic error by measuring the frequency split of the *n* = 1 mode.

Table 2 presents an overview of the qualitative impact of the harmonic errors of the imperfect density on the frequency splits of the three modes. The second harmonic error can be identified by the frequency split Δ*f*_1_. However, there is no significant difference of the first three harmonic errors on the frequency split of the *n* = 2 and the *n* = 3 modes. The relations between the harmonic errors and the frequency split are also listed. There is a linear relation between the second harmonic error and the frequency split Δ*f*_1_. The third harmonic error makes no significant impact on the frequency split of the *n* = 1 and the *n* = 2 mode. By contrast, the relation of the rest is quadratic or quadratic like.

## 4. Method of Reduction of the Second Harmonic Error

### 4.1. Chemical Balancing of the Second Harmonic Error

The chemical balancing procedure utilizes hydrofluoric acid to remove mass from fused silica resonators, which was first proposed by Basarab et al. [27,34] and extended in our previous work [30]. However, these studies have emphasized the chemical balancing on the fourth harmonic errors. Although Basarab et al. had presented brief examples for the treatment of other harmonic errors, the principles and properties of the chemical balancing on the second harmonic error need further investigation. In particular, the removal of the *k*-th harmonic error results in the generation of the *ik*-th (*i* = 2, 3, 4…) harmonics. This means the change in the second harmonic error also alters the fourth harmonic error, which may result in a larger frequency split of the *n* = 2 mode and dramatically degrade the performance of the resonator. Fortunately, by carefully selecting the chemical balancing parameters, it is possible to reduce the second harmonic error without introducing new harmonic errors. 

Although the basic chemical trimming theory and its application on the fourth harmonic error balancing has been built and analyzed in our previous work [30], the properties of the chemical trimming theory in the second harmonic error case have not been illustrated and verified on cylindrical resonators. Therefore, we make efforts to figure out the relationship between the balancing mass and the balancing parameters. 

**Figure 8:**
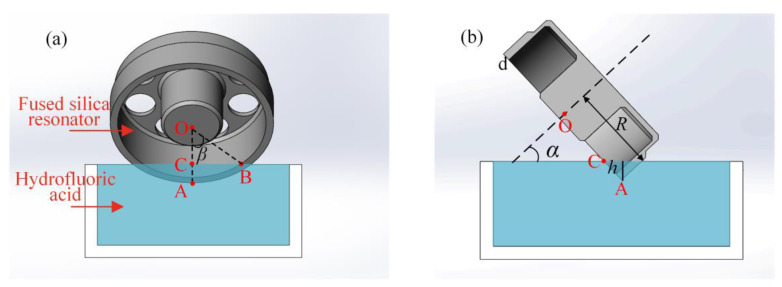
Schematic view of the chemical balancing: (**a**) The front view; (**b**) The half-section view.

As shown in Figure 8, the cylindrical fused silica resonator is partially soaked in the hydrofluoric acid at inclined angle *α*. Angle *β* is the included angle of the line segment OA and OB. *R* is the inner radius of the resonator shell, *d* is the thickness of the resonator shell, and *h* is the immersed depth of the resonator. Point O is the center of the resonator top surface. Point A is the lowest position of the immersed part, and point C is the intersection point of the line segment OA and the liquid level.

According to the multi-mode trimming theory [35,36,37], the second harmonic error could be balanced by removing mass from two positions starting from the heavy axis where the value of the second harmonic error reaches maximum. The mass element removed by chemical balancing from the two positions can be express as:

(9) $$dm=2v\rho (C_{0}\left(\alpha ,\beta \right)+C_{2}\left(\alpha ,\beta \right)\cos2\varphi +C_{4}\left(\alpha ,\beta \right)\cos4\varphi )d\varphi ,$$

where *v* is the etching rate of the hydrofluoric acid on fused silica in μm/min, *C*_0_(*α*, *β*), *C*_2_(*α*, *β*), *C*_4_(*α*, *β*) are the coefficients of Fourier series given in [30], and the coefficients higher than four is omitted in this case.

**Figure 9:**
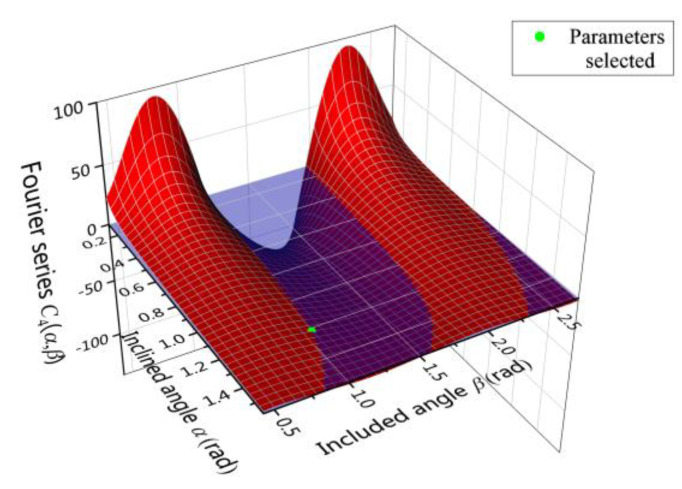
Variation of the *C*_4_(*α*, *β*) with inclined angle *α* and included angle *β*.

As Equation (9) suggests, the removal of the second harmonic error could lead to the variation of the fourth harmonic error. However, it is possible to select the inclined angle *α* and the included angle *β* such that *C*_4_(*α*, *β*) = 0. Under this condition, the existing fourth harmonic error is immune to the balance of the second harmonic error. The desired parameters *α, β* lie on the intersecting lines of the zero-value surface in blue and the curved surface in red, as shown in Figure 8. There are three intersecting lines with numerous combinations of *α* and *β*. The larger angle *β* means the deeper immersed depth during chemical balancing, and the larger inclined angle *α* means that the resonator is more perpendicular to the liquid level. Taking both the experimental apparatus and the resonator structure into consideration, the angle *α, β* is finally set as 1.22 rad and 1.05 rad, which is marked green in Figure 9.

In the practical balancing process, the resonator is etched at inclined angle *α* and immersed depth *h*. According to balancing setup shown in Figure 7, the immersed depth can be expressed as:

(10) h=(R−Rcosβ)cosα.

Taking the selected parameters *α* and *β* into Equation (10), the immersed depth *h* is 2.25 mm in this case.

### 4.2. Determination of the Trimming Positions

One crucial problem to be solved is the determination of trimming positions when balancing the second harmonic error. It has been demonstrated that the trimming positions of the fourth harmonic error should be located at the principal axis of the lower natural frequency of the *n* = 2 mode [38]. However, the trimming positions of the *n* = 1 mode has rarely been discussed in the open literature. In this section, simulation analysis is presented for investigation of the trimming positions of the *n* = 1 mode. 

**Figure 10:**
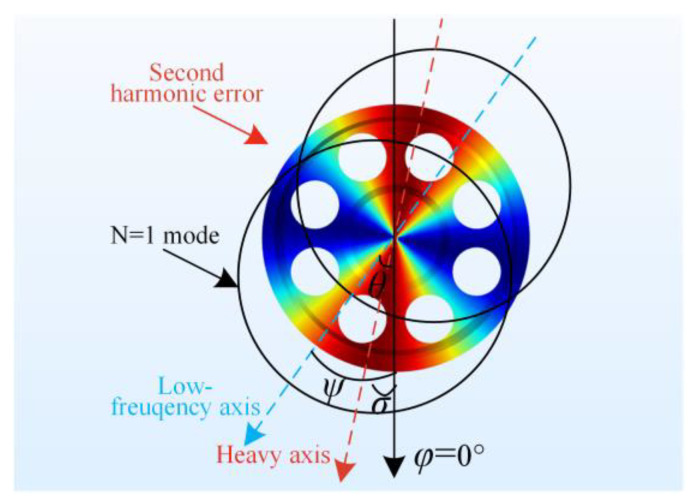
The low-frequency axis of the *n* = 1 mode and the heavy axis of the second harmonic error.

As shown in Figure 10, angle *σ* is the circumferential location of the heavy axis, where the second harmonic error reaches the maximum. Angle *ψ* is the circumferential location of the low-frequency axis of the *n* = 1 mode, where the natural frequency reaches the minimum. Angle *θ* is the intersecting angle between the low-frequency axis and the heavy axis. We define the clockwise direction as the positive direction.

**Figure 11:**
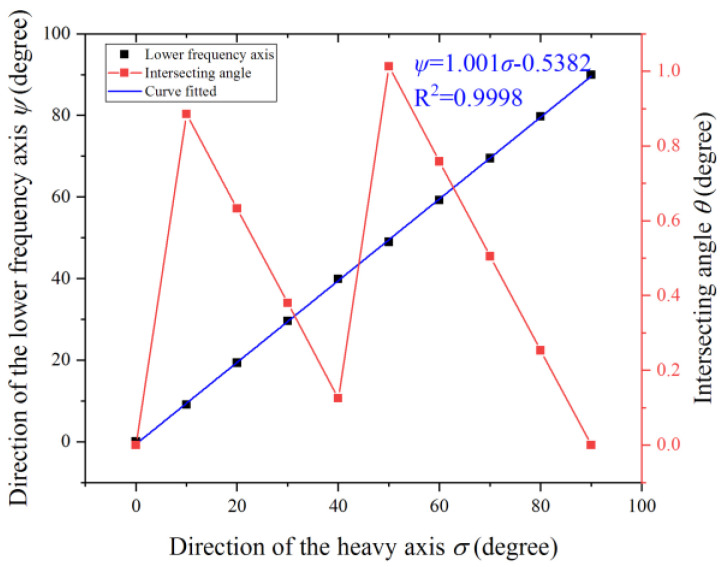
Relation between the heavy axis and the low-frequency axis of the *n* = 1 mode.

The relation between the angle *ψ* and *σ* is demonstrated by varying the angle *σ* and then locating the low-frequency axis. Figure 11 illustrates that the low-frequency axis coincides with the heavy axis. The angle of the low-frequency axis of the *n* = 1 mode is proportional to the angle of the heavy axis with a slope of 1.001. The intersecting angle *θ* ranges from 0 to 1 degree when *σ* increases from 0 to 90 degrees. These slight variations of the intersecting angle *θ* mainly stem from simulation errors. Therefore, the defect mass could be removed from the direction of the low-frequency axis.

## 5. Results and Discussion

**Figure 12:**
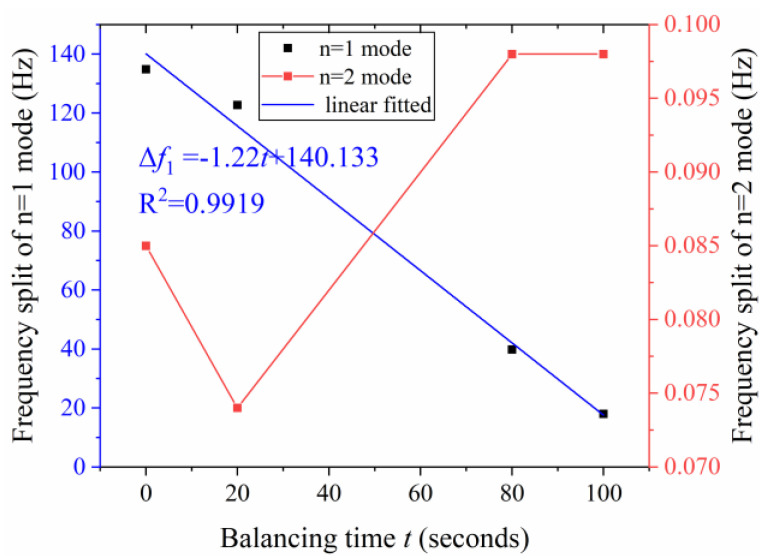
Variations of frequency split of the *n* = 1 mode and the *n* = 2 mode during the three rounds chemical balancing on resonator R01.

Chemical balancing experiments on two cylindrical fused silica resonators are performed. The frequency split and the direction of the low-frequency axis of the *n* = 1 mode are measured by the laser Doppler vibrometer (PSV-500). The resonator is excited by a loudspeaker. The axial vibration of the resonator shell is measured by the laser Doppler vibrometer. The natural frequency axis locates at the direction where the axial vibration reaches the maximum. Then, frequency sweeping is conducted in these directions. The direction of higher frequency is the high-frequency axis. The direction of lower frequency is the low-frequency axis. The frequency split is obtained by the frequency sweeping at the location which is the 22.5° (*n* = 2) or 45° (*n* = 1) apart from the low-frequency axis. The natural frequencies and frequency split of the *n* = 1 and *n* = 2 modes are measured respectively. The resonator is then transferred to the chemical balancing system. The inclined angle *α* and the immersed depth *h* are set as 1.22 rad and 2.25 mm according to theoretical analysis. The balancing time *t* depends on the etching rate *v* and the residual frequency split of the *n* = 1 mode. The resonator is lifted by the electronic platform after being etched for *t*/2 period, and is rotated 180 degrees for another *t*/2 period at the next trimming position.The detailed balancing process on resonator R01 is shown in Figure 12. Three rounds of chemical balancing experiments are performed on resonator R01. The frequency split Δ*f*_1_ linearly decreases with the total balancing time *t*. The frequency split Δ*f*_2_ ranges from 0.075 Hz to 0.098 Hz during the balancing process.

**Figure 13:**
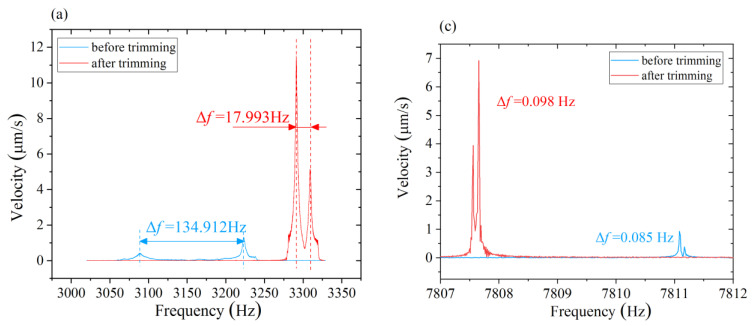

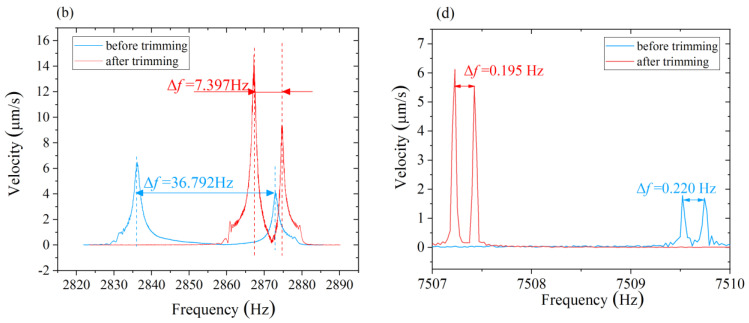
Variations of frequency split before and after chemical balancing: (**a**) *n* = 1 mode of Resonator R01; (**b**) *n* = 1 mode of Resonator R02; (**c**) *n* = 2 mode of Resonator R01; (**d**) *n* = 2 mode of Resonator R02.

As shown in Figure 13a, the initial natural frequency of the *n* = 1 mode of resonator R01 is 3223.438 Hz and 3088.526 Hz, respectively, with an initial frequency split of up to 134.912 Hz, which implies the second harmonic error of resonator R01 is large. After several rounds of chemical balancing, the lower natural frequency increases to 3291.431 Hz and the higher natural frequency increases to 3309.424 Hz. The frequency split of the *n* = 1 mode decreases to 17.993 Hz with a drop of 86.6% compared to the original frequency split. Meanwhile, the vibration velocity at the low-frequency axis increases markedly from 0.433 μm/s to 11.54 μm/s, and the vibration velocity at the high-frequency axis increases from 1.327 μm/s to 5.16 μm/s. 

Experimental results on another same-structure resonator R02 are shown in Figure 13b. The initial natural frequency of resonator R02 is 2872.925 Hz and 2836.133 Hz, respectively, with an initial frequency split of 36.792 Hz. After one round of chemical balancing, the lower natural frequency increases to 2874.658 Hz and the higher natural frequency increases to 2867.261 Hz. The frequency split of the *n* = 1 mode reduces to 7.397 Hz with a drop of 79.8%. Meanwhile, the velocity of vibration at the high-frequency axis increases from 4.22 μm/s to 9.36 μm/s. The velocity of vibration at the low-frequency axis increases from 6.49 μm/s to 14.88 μm/s. 

The reason for the dramatic increase in the velocity of vibration could be that, as the second harmonic error decreases, the vibration of the resonator is also less coupled with the base, resulting in less energy dissipation from the base. Meanwhile, the etching rate *v* remains constant during the balancing experiments. Hence, the mass etched per unit time is the same, which means that the frequency split of the *n* = 1 mode is proportional to the second harmonic error of the defect mass. The linear relation between the frequency split of *n* = 1 mode and the etched mass obtained from balancing experiments is consistent with the theoretical calculation.

The natural frequency and frequency split of the two resonators before and after chemical balancing are shown in Figure 13c,d. The natural frequency of the *n* = 2 mode decreased after chemical balancing. It is noticed that, although we chose a set of balancing parameters for the second harmonic error that theoretically has no impact on the fourth harmonic error, Δ*f*_2_ still has a slight change with the balancing of the second harmonic error. The fluctuation of the Δ*f*_2_ mainly stems from the following two aspects: one is the inevitable error during the measurement of the low-frequency axis of the *n* = 1 mode. Meanwhile, the control of the inclined angle, immersed depth, and rotating angle may have a slight deviation from the ideal value. These possible sources of error could have affected the fourth harmonic error of the resonator. However, the maximum variation of the Δ*f*_2_ during the balancing of is about 0.024 Hz, which indicates that the change the fourth harmonic error is quite small during the removal of the second harmonic error under the selected balancing parameters where inclined angle *α* is 1.22 rad and the immersed depth *h* is 2.25 mm. 

The experimental results on resonator R01 and R02 illustrate that the second harmonic error can be reduced from the direction of the low-frequency axis, and the frequency split of the *n* = 1 mode can be a reference to the residual value of the second harmonic error. The velocity of the vibration is significantly increased which benefits from the decline of the second harmonic error. The performance of the resonator is further improved after balancing on the second harmonic error. The balancing method of the second harmonic error provides an effective and reliable guidance in the determination of the balancing parameters on resonators with different structures. 

## 6. Conclusions

In this paper, a new method to identify and reduce the second harmonic error is proposed and verified. The results of the theoretical calculation clearly indicate that the frequency split of the *n* = 1 mode is proportional to the second harmonic error. The simulations have identified the effects of the first three harmonic error on the frequency splits. The second harmonic error makes the major contribution to the frequency split of the *n* = 1 mode. The second harmonic error can be calculated from the linear relation on the Δ*f*_1_. The chemical balancing parameters are specially selected to remove the second harmonic error without affecting other harmonics. The second harmonic error of resonator R01 and R02 is independently reduced by 86.6% and 79.8% when the inclined angle α is 1.22 rad and the immersed depth is 2.25 mm. Both the simulation and experimental result confirms the linear relation between the frequency split of *n* = 1 mode and the second harmonic error. This paper provides an effective and convenient approach to measure and reduce the second harmonic error, by which the performance of the cylindrical resonator gyroscope can be further improved.

## Figures and Tables

**Table 1:** Material and structure parameters of the resonator.

Perfect density	2203 kg/m^3^
Young’s modulus	71.7 GPa
Poisson’s ratio	0.17
Inner radius of resonator shell	12 mm
Thickness of resonator shell	1.2 mm

**Table 2:** Contribution of the first three harmonic errors on each mode when *ρ_i_* = 100 kg/m^3^ and the relations between the frequency splits and the first three harmonic errors.

Mode		The First Harmonic Error	The Second Harmonic Error	The Third Harmonic Error
*n* = 1	Contribution	1.35%	98.51%	0.14%
	Relation	Quadratic	Linear	Nearly no impact
*n* = 2	Contribution	0.24%	99.45%	0.31%
	Relation	Approximate Quadratic	Quadratic	Nearly no impact
*n* = 3	Contribution	3.49%	83.95%	12.56%
	Relation	Quadratic	Approximate Quadratic	Quadratic
